# The complete genome sequence of unculturable *Mycoplasma faucium* obtained through clinical metagenomic next-generation sequencing

**DOI:** 10.3389/fcimb.2024.1368923

**Published:** 2024-04-16

**Authors:** Artur J. Sabat, Tim Durfee, Schuyler Baldwin, Viktoria Akkerboom, Andreas Voss, Alexander W. Friedrich, Erik Bathoorn

**Affiliations:** ^1^ Department of Medical Microbiology and Infection Prevention, University Medical Center Groningen, University of Groningen, Groningen, Netherlands; ^2^ DNASTAR, Inc., Madison, WI, United States; ^3^ University Hospital Münster, University of Münster, Münster, Germany

**Keywords:** *Mycoplasma faucium*, metagenomics, lung transplant patient, insertion sequence, ice

## Abstract

**Introduction:**

Diagnosing *Mycoplasma faucium* poses challenges, and it's unclear if its rare isolation is due to infrequent occurrence or its fastidious nutritional requirements.

**Methods:**

This study analyzes the complete genome sequence of *M. faucium*, obtained directly from the pus of a sternum infection in a lung transplant patient using metagenomic sequencing.

**Results:**

Genome analysis revealed limited therapeutic options for the *M. faucium* infection, primarily susceptibility to tetracyclines. Three classes of mobile genetic elements were identified: two new insertion sequences, a new prophage (phiUMCG-1), and a species-specific variant of a mycoplasma integrative and conjugative element (MICE). Additionally, a Type I Restriction-Modification system was identified, featuring 5’-terminally truncated *hsdS* pseudogenes with overlapping repeats, indicating the potential for forming alternative *hsdS* variants through recombination.

**Conclusion:**

This study represents the first-ever acquisition of a complete circularized bacterial genome directly from a patient sample obtained from invasive infection of a primary sterile site using culture-independent, PCR-free clinical metagenomics.

## Introduction

In 1969, *Mycoplasma faucium* was first reported and initially considered a rare commensal inhabitant of humans, originally naming it *Mycoplasma orale* type 3 (Fox et al., 1969). The cells of *M. faucium* primarily exhibit a coccoidal shape, although they can also manifest in coccobacillary and very short filamentous forms (Arabatzis and Velegraki, 2022). Phylogenetic analysis, relying on partial 16S rRNA gene sequences, revealed that *M. faucium* belongs to the *M. hominis* cluster (Rawadi et al., 1998). Members of the *M. hominis* cluster exhibit significant biochemical similarities, such as the ability to perform arginine hydrolysis and the inability to ferment glucose (Pettersson et al., 2000). *M. faucium* typically resides in the human oropharynx as a commensal organism but can potentially disseminate to other parts of the body. It has been found in the skin lesions of psoriatic patient as well as in a case of skin abscess in patient with activated phosphoinositide 3-kinase δ syndrome type 2 (APDS2) (Dominguez-Pinilla et al., 2018; Arabatzis and Velegraki, 2022). The presence of *M. faucium* has been identified in gastric tissue samples obtained from Korean chronic gastritis patients (Kwon et al., 2004). While *M. faucium* in gastric tissues might be considered as contaminants from the oropharynx, the higher frequency of *M. faucium* compared to *M. salivarium* and *M. orale* in chronic gastritis tissues contradicts this assumption. *M. salivarium* is typically detected in 60–80% of throat specimens from adults, *M. orale* in 30–60%, whereas *M. faucium* is infrequently isolated from the human oropharynx (Tully, 1993). Mycoplasmas present in tissue samples from chronic gastritis patients exhibit similarities to *Helicobacter pylori*. Non-fermentative Mycoplasmas such as *M. faucium*, which produce ammonia from arginine can cause tissue damage and neutralize gastric acid (Kwon et al., 2004). Furthermore, *M. faucium* has been associated with brain abscess in a non-immunocompromised patients (Al Masalma et al., 2009; Rothman et al., 2022).

The diagnosis of *M. faucium* poses challenges, as this bacterium is unculturable in standard media. It remains unclear whether its rare isolation is due to infrequent occurrence or linked to its fastidious nutritional requirements. To date, the NCBI database contains only partial sequences of *M. faucium* 16S rRNA, 23S rRNA and *rpoB* genes. In this study, we present comprehensive analysis of the complete closed *M. faucium* genome sequence, which was obtained directly from the pus of a sternum infection in a lung transplant patient through metagenomic next-generation sequencing.

## Materials and methods

### Extraction of genomic DNA

The pus sample was homogenized with a TissueLyser II (Qiagen, Germantown, MD, USA) and total DNA was purified using the DNeasy Blood & Tissue Kit (Qiagen, Hilden, Germany). DNA was quantified using a Qubit 2.0 fluorometer (ThermoFisher Scientific, Waltham, Massachusetts, US) and the quality was assessed by TapeStation 2200 (Agilent Technologies, Santa Clara, California, US). A NanoDrop 2000c spectrophotometer (ThermoFisher Scientific) was used to measure the purity of extracted DNA.

### Bacterial species identification

Pus collected from the substernal region of a lung transplant patient with sternomediastinitis was used for direct microscopic examination, as well as bacterial and fungal culture. Fifty nanograms of total DNA extracted from the pus sample was amplified with 0.5 μM universal bacterial primers LPW57 (5′-AGTTTGATCCTGGCTCAG-3′) and LPW58 (5′-AGGCCCGGGAACGTATTCAC-3′) using Platinum SuperFi PCR Master Mix (Invitrogen). PCR amplification was performed on a PTC-100 thermocycler (Bio-Rad) using the following conditions: an initial incubation at 98°C for 30 s, 40 cycles of 98°C for 10 s, 58°C for 10 s, and 72°C for 45 s, followed by a final incubation at 72°C for 5 min. Size and purity of the amplified DNA fragment was estimated with a 2200 TapeStation using the Agilent D5000 ScreenTape and Reagens according to the manufacturer’s instructions. PCR product was purified using DNA Clean & Concentrator (Zymo Research). DNA Sanger sequencing was performed using the PCR primers. The amplified nucleotide sequence of the 16S rRNA was then compared to sequences available in GenBank using the blastn algorithm.

### Whole-genome sequencing, assembly, and annotation

Illumina genomic libraries were sequenced on a NovaSeq 6000 system with a 2 x 150 paired-end protocol. Oxford Nanopore sequencing libraries were prepared with the Rapid Barcoding kit and sequenced on a MinION device using flow cell type R9.4.1. Nanopore reads were first aligned to the human GRCh38 reference genome using SeqMan NGen version 18 (DNASTAR, Madison, Wisconsin, US) running in templated assembly mode. Unaligned reads corresponding to the non-human content of the sample were *de novo* assembled with SeqMan NGen assembler running in *de novo* mode and Canu (Koren et al., 2017). Genome finishing was done with Nanopore and Illumina datasets using automated workflows in SeqMan NGen and manual editing in SeqMan Pro (DNASTAR). Genome alignments were done with Mauve (Darling et al., 2004) running within MegAlign Pro (DNASTAR). The complete genome sequence was annotated by the NCBI Prokaryotic Genome Annotation Pipeline (PGAP).

### Phylogenetic analysis based on the 16S rRNA gene sequences

The entire 16S rRNA nucleotide sequences were aligned with Clustal Omega implemented in the MegAlign software version 18 (DNASTAR). Adding to the newly sequenced *M. faucium* UMCG-MFM1, the phylogenetic tree included additional 16S rRNA gene sequences extracted from publicly available annotated genomes at the NCBI database representing 42 *Mycoplasma* species. Ureaplsma parvum species served as the outgroup. Phylogenetic tree was reconstructed based on the maximum likelihood (ML) method and 1000 bootstrap replications using the MegAlign software Pro v. 18 (DNASTAR).

### Phylogenetic analysis based on concatenated conserved proteins

Identification of single-copy protein-encoding orthologs, which are genes with a common ancestral origin and that are universally present across a group of selected organisms was performed using an ortholog finding algorithm in SeqMan NGen. Candidates that exceeded Smith-Waterman pairwise protein alignment thresholds were concatenated into single sequences for each *Mycoplasma* examined and a multiple sequence alignment (MSA) generated with the MAFFT alignment algorithm in MegAlign Pro. Phylogenetic trees were reconstructed from the MSA using the RAxML maximum likelihood method with 1000 bootstrap replications in MegAlign Pro.

### Horizontal gene transfers

Potential horizontal gene transfers (HGTs) were identified according to the methodology outlined in a previous study (Pereyre et al., 2009), with some minor modifications. For each predicted protein in the *M. faucium* UMCG-MFM1 genome, the best BLAST hits (BBHs) were detected using a BLASTp threshold E-value of 10^-8^. CDSs exhibiting a BBH from a species outside the *M. hominis* cluster were further investigated by phylogenetic analyses. Protein sequences were aligned in MegAlign Pro module version 18 (DNASTAR) using the MAFFT method. The aligned sequences were exported as a FASTA file. Protein phylogenies were established using the MEGA11 software (Tamura et al., 2021). Tree construction employed the Maximum Likelihood method with bootstrap statistical analyses conducted with 500 replicates, and bootstrap values below 90% were not considered significant. In instances where phylogenetic congruence existed between a protein sequence deduced from the *M. faucium* genome and those retrieved from the *M. hominis* cluster, supported by significant bootstrap values, they were excluded as potential HGT events. Conversely, proteins exhibiting incongruence between the phylogenies of *M. faucium* and species within the *M. hominis* cluster, supported by significant bootstrap values, were considered as potential candidates for HGT events. Moreover, cases where incongruence was observed between protein and species phylogenies, with branches lacking significant bootstrap values and corresponding homologs within the *M. hominis* cluster could not be identified in the NCBI database, were also considered as potential candidates for HGT events.

### Data analysis

The presence of acquired antimicrobial-resistance genes was assessed using Res-Finder 4.1 (https://cge.food.dtu.dk/services/ResFinder/) and CARD (The Comprehensive Antibiotic Resistance Database, https://card.mcmaster.ca/). Potential subpopulation elements were searched for using a SeqMan NGen templated assembly of the Illumina data evaluated with a simple SNV caller. The average nucleotide identity (ANI) values among *Mycoplasma* species were calculated using JSpecies Web Server (JSpeciesWS) based on BLAST alignments (with ANIb method) (Richter et al., 2016). PHASTER (PHAge Search Tool Enhanced Release) was used to analyze prophages in the genome (Arndt et al., 2016). Direct and inverted repeats were identified by the GenQuest module version 18 (DNASTAR). Prediction of Signal Peptides was achieved with SignalP-6.0 server (https://services.healthtech.dtu.dk/services/SignalP-6.0/). DeepTMHMM scan was applied to search for the presence of transmembrane domains (https://dtu.biolib.com/DeepTMHMM). The Phyre2 web portal was used for protein modelling, prediction and analysis (Kelley et al., 2015). Virulence genes were searched using VFDB (http://www.mgc.ac.cn/VFs/): a reference database for bacterial virulence factors.

### Data availability

The complete sequence of the chromosome of *M. faucium* UMCG-MFM1 and raw sequencing reads have been deposited in the GenBank database and are available under the Bioproject PRJNA783792.

## Results

### Bacterial species identification

A sample from a pus pocket in the sternum was perioperatively taken in a patient six months after lung transplantation. In this sample, no microorganism was visualized through microscopy, and both aerobic and anaerobic cultures showed no growth. PCR amplification of the 16S rRNA gene from a DNA sample prepared from the pus sample produced an amplicon of the expected size, approximately 1.4 kb. Sanger sequencing of the amplicon revealed that the partial 16S rRNA gene sequence shared the highest nucleotide identity with the 16S rRNA gene sequence from *M. faucium* strain DC333 (99.70%), followed by *Mycoplasma orale* strain NCTC10112 (97.88%) as the second best species. Following CLSI guidelines, where a species assignment is made when the maximum score is 99% or higher, and the sequence similarity between the best and second-best species is more than 0.5% (Sabat et al., 2017), we concluded the sternum of the transplant patient was infected with *M. faucium*. Histologic examination of a sternal bone fragment confirmed that there was an active osteomyelitis. In an attempt to culture the *M. faucium*, pleuropneumonia-like organisms agar plates were incubated for up to one month at 35°C under aerobic and anaerobic conditions. No colonies were visible by plate microscopy.

### Genomic DNA sequencing and annotation of *M. faucium* UMCG-MFM1

Using the Oxford Nanopore platform, we generated a total of 5,488,683 reads from the patient sample, resulting in a total yield of 4.81 Gbases with an average quality score of 12.8. Among them, 1,566,520 reads exceeded 1,000 nucleotides, with the longest read measuring 87,188 nucleotides. The entire dataset was first aligned to the human GRCh38 reference genome to remove “contaminating” reads from the patient’s genome. Unaligned reads representing the non-human content of the sample were *de novo* assembled using the SeqMan NGen and Canu assemblers. Both assemblers produced a single large contig in the expected size range (~800kb) sequenced to a depth of ~100x that shared >99.9% sequence identity. Alignment of paired end Illumina data to the consensus sequence from either assembly indicated the sequences were topologically correct except for an unresolved tandem duplication present in both along with a spurious duplication in the Canu generated sequence. The SeqMan NGen consensus was the basis for resolving the duplication and producing a final polishing sequence using a combination of the Nanopore and Illumina datasets. The complete *M. faucium* UMCG-MFM1 genome consisted of a single 806,567 bp circular chromosome. The two copies of the tandem duplication were 17,372 bp (genome coordinates 129,844-147,215) and 16,168 bp (genome coordinates 147,216-163,383) in length and shared >99.9% sequence identity. No plasmids were detected in the genome.

To assess if there could be another microorganism in the patient sample, Nanopore reads were mapped against either the human reference genome GRCh38 sequence or the *M. facium* genome sequence obtained in this study. Among the 5,488,683 total reads, 5,426,772 aligned to GRCh38 (98.87%), 32,144 aligned to *M. facium* (0.59%), and 29,767 were unaligned (0.54%). Running the unaligned reads against the FDA-ARGOS (Sichtig et al., 2019) or MBGD (Uchiyama et al., 2019) microbial genome databases did not yield significant matches. Additionally, *de novo* assembly resulted in only 2 small contigs, and these did not show significant matches against the NCBI nr (non-redundant) database. Therefore, we concluded that there was not another microorganism in the patient sample and the unaligned reads did not assemble likely because of poor quality.

The overall G+C content was 24.17% and 729 genes were identified in total (**Table 1**). Among the 683 protein-coding genes, 273 (40.0%) were classifiable as enzymes, including 50 transferases, 24 nucleases, 22 ligases, 17 transposases, 16 peptidases, 15 permeases, 13 kinases and 12 ATPases. There were also 234 CDSs (34,3%) annotated as “hypothetical proteins” of unknown function.

**Table 1:** Gene content of the chromosome of *M. faucium* UMCG-MFM1.

Features annotated	Number of features
Genes (total)	729
Coding DNA sequences (total)	690
Coding DNA sequences (with protein)	683
Genes (RNA)	39
rRNAs (5S, 16S, 23S)	1, 1, 1
tRNAs	33
ncRNAs	3
Pseudogenes	7

### Phylogenetic analysis

**Figure 1:**
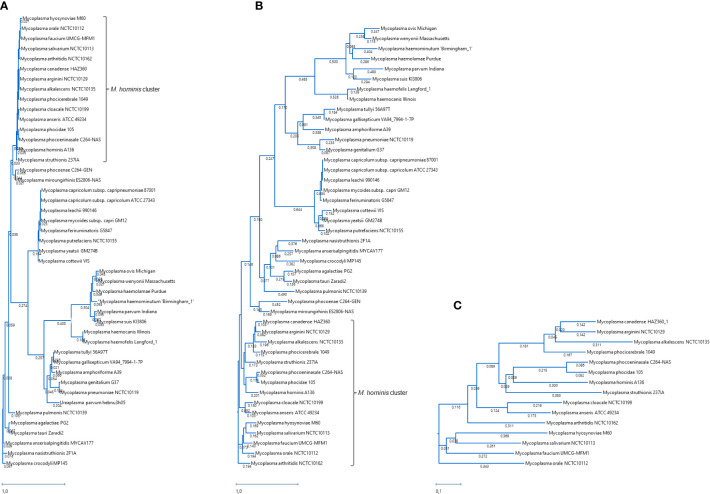
Phylogenetic trees based on three different sets of genes: **(A)** entire 16S rRNA genes, **(B)** 11 genes present in all analyzed *Mycoplasma* species, and **(C)** 109 genes present in all species of the hominis cluster. The trees were constructed using the Maximum Likelihood method by comparing nucleotide sequences **(A)** or translated amino acid sequences of the universal *Mycoplasma* genes **(B)** or ubiquitous genes of the hominis cluster **(C)** generated with the MegAlign Pro module (DNASTAR). All branch lengths are drawn to a scale bar, reflecting evolutionary distances equivalent to one replacement per every 100 nucleotides or amino acids.

To show the evolutionary relationships and genetic relatedness of *M. faucium* among different *Mycoplasma* species, a phylogenetic tree was constructed based on complete 16S rRNA nucleotide sequences. This analysis placed *M. faucium* within the *M. hominis* cluster (**Figure 1A**), consistent with previous investigations based on partial 16S rRNA nucleotide sequences (Rawadi et al., 1998). Within the *M. hominis* cluster, nucleotide identity ranged from 95.72% to 99.63%. Notably, the 16S rRNA gene sequence of *M. faucium* exhibited the highest similarity to *M. orale*, *Mycoplasma hyosynoviae*, and *Mycoplasma salivarium*, with similarity values of 98.23%, 98.01%, and 97.64%, respectively.

We identified eleven single-copy universally present protein homologs in the *Mycoplasma* genus (**Supplementary Table S1**). Phylogenetic comparison of these protein sequences largely mirrored the species clustering observed in the 16S rRNA analysis (**Figure 1B**). However, *M. faucium* displayed the highest similarity to *M. salivarium* (82.74%), followed by *M. hyosynoviae* (82.42%) and *M. orale* (81.57%).

Phylogenies based on the *M. hominis* cluster provided enhanced resolution at the narrow species level (**Figure 1C**). Within the cluster, we identified 109 ubiquitous homologs (**Supplementary Table S2**) and subsequently used their protein sequences for phylogenetic analysis. In this context, *M. faucium* exhibited the highest similarity to *M. salivarium*, with a similarity value of 71.67%. Notably, when compared to the second closest species, *M. orale* (69.89%), and the third closest species, *M. hyosynoviae* (69.10%), the differences in similarity between *M. salivarium*, *M. orale*, and *M. hyosynoviae* were more pronounced than those observed for the nine *Mycoplasma* universal proteins.

### Average nucleotide identity

The results of the ANI analysis on the *Mycoplasma* strains further validated species identification. High-throughput ANI analysis of 90,000 prokaryotic genomes revealed clear species boundaries (Jain et al., 2018). This analysis showed that ANI values exceed 95% within species and fall below 83% between different species (Jain et al., 2018). Due to the limitation of the JSpecies Web Server, which can only handle 30 genomes at a time, 44 genomes were split into two analyses. In the first analysis, *M. faucium* was compared only to species within the *M. hominis* cluster, while in the second analysis *M. faucium* was compared only to species outside the *M. hominis* cluster. The ANI values between UMCG-MFM1 and other *Mycoplasma* species ranged between 70.33% and 73.56 % within the *M. hominis* cluster (**Supplementary Table S3**) and between 62.53% and 68.96% compared to species outside the *M. hominis* cluster (with the exception of *Mycoplasma miroungirhinis*, 71.06%) (**Supplementary Table S4**). These findings strongly indicated that UMCG-MFM1 represented a distinct species within the *M. hominis* cluster.

### Antibiotics

Tetracyclines, fluoroquinolones, and macrolides are the most potent anti-mycoplasma agents. Given the impracticality of routine laboratory cultivation and antibiotic Minimum Inhibitory Concentration (MIC) determination, the prediction of antibiotic resistance through genome sequence analysis emerges as the sole practical option in *M. faucium* infections.

*Mycoplasma* resistance to tetracycline is linked to the acquisition of the *tet*(M) gene via transposons and/or plasmids (Chalker et al., 2021). Additionally, mutations in the tetracycline-binding unit of 16S rRNA, such as those at positions G346A, A965T, and G966T (*Escherichia coli* numbering), can lead to reduced susceptibility to this antibiotic (Dégrange et al., 2008; Amram et al., 2015). However, ResFinder and CARD analyses did not detect any acquired antimicrobial resistance genes, and sequence analysis of the 16S rRNA gene revealed no mutations associated with tetracycline resistance. Therefore, *M. faucium* UMCG-MFM1 is possibly susceptible to tetracycline.

Bacterial resistance to fluoroquinolones is attributed to amino acid alterations in DNA gyrase (GyrA and GyrB) and/or the topoisomerase IV complex (ParC and ParE) (Drlica and Zhao, 1997). Sequence analysis revealed *gyrA* and *parC* were wild-type, but that *parE* contained a non-synonymous substitution resulting in an alanine to serine change at position 463 (A463S). This ParE A463S mutation was previously reported in two *M. hominis* strains, typically in association with GyrA S153L (Zhang et al., 2019). In those strains, the presence of ParE A463S was found to impact the MIC of levofloxacin (LVX), raising it from 8-64 to 128 µg ml^−1^ compared to strains lacking this substitution.

Macrolides are the most effective anti-mycoplasma agents. However, *M. hominis* exhibits intrinsic resistance to 14- and 15-membered macrolides, including erythromycin. In *M. hominis*, this resistance is linked to a guanine-to-adenine transition at position 2057 (G2057A; *E. coli* numbering) in domain V of 23S rRNA (Furneri et al., 2000). This mutation disrupts macrolide binding to the ribosome. Sequence comparison showed that the 23S rRNA gene of *M. faucium* contained the same mutation, suggesting a potential mechanism for macrolide resistance.

Finally, *M. faucium* harbors an amino acid substitution H526N (*E. coli* numbering) in the RNA polymerase beta-subunit (RpoB), indicating resistance to rifampin, consistent with observations in other *Mycoplasma* species (Gaurivaud et al., 1996).

To search for potential single nucleotide variants (SNVs) and/or small indels in known resistance genes present in subpopulations of the sequenced sample, a templated assembly of the Illumina data onto the *M. faucium* genome (median depth of coverage = 68.5x) was evaluated for positions with a secondary base occurring in at least 5% of the reads. No non-synonymous changes in protein encoding genes or SNVs at positions known to confer resistance in non-coding RNA genes, such as 16S rRNA or 23S rRNA, were identified indicating the absence of such elements in the infecting population.

### Mobile genetic elements

Three different classes of mobile genetic elements (MGE) were identified in the *M. faucium* genome: (i) two new insertion sequences (ISs), (ii) a new prophage, phiUMCG-1, and (iii) a species-specific variant of a mycoplasma integrative and conjugative element (termed MICE) encoding a T4SS secretion system.

### Insertion sequences

Two novel ISs were identified on the UMCG-MFM1 chromosome. The first IS, designated ISMfau-1, was identified in seven copies (**Supplementary Table S5**) each of which carried only a single open reading frame (ORF) of 423 amino acids encoding the transposase TnpA. BLAST searches indicated that the encoded protein had the highest identity (69.7%) with the transposase of the IS3 family, previously found in the *M. hyosynoviae* genome. The *tnpA* gene was surrounded by nontranslated regions: 61-62 bp at the 5’- end and 4 bp at the 3’-end. The ISMfau-1 element was flanked by inverted repeats (IRs), 5’-TAAACTWGGACAAA-3’ and 3’-ATTTGATCCTGTTT-5’ at its 5’ and 3’ ends, respectively. The complete ISMfau-1 consisted of 1365-1366 bp, including inverted repeats. A distinctive feature of ISMfau-1 was that transposition did not result in the creation of target site duplications.

The second IS, designated ISMfau-2, was discovered in ten copies (**Supplementary Table S5**). Interestingly, unlike ISMfau-1, the majority of ISMfau-2 copies (nine out of ten) were accompanied by distinct individual direct repeats, each with unique sequence specific to the locus. Furthermore, the length of these direct repeats varied from 19 to 29 bp at different loci. The ISMfau-2 element itself had a length ranging from 1252 to 1256 bp, between its corresponding direct repeats. Similar to ISMfau-1, ISMfau-2 contained a single ORF of 339 amino acids that displayed the highest similarity (59.5%) to the transposase of the IS30 family previously identified in the *M. orale* genome. ISMfau-2 had perfect terminal inverted repeats of 15 bp: 5’-CATAGTGTTGCACTT-3’ (located 67 or 69 nucleotides before the start codon of the *tnpA* gene) and 3’-GTATCACAACGTGAA-5’ (located 103-105 nucleotides after the stop codon). Direct and inverted repeats were separated between each other by non-repeated 16 bp sequences belonging to the ISMfau-2 element.

### Prophage phiUMCG-1

**Figure 2:**
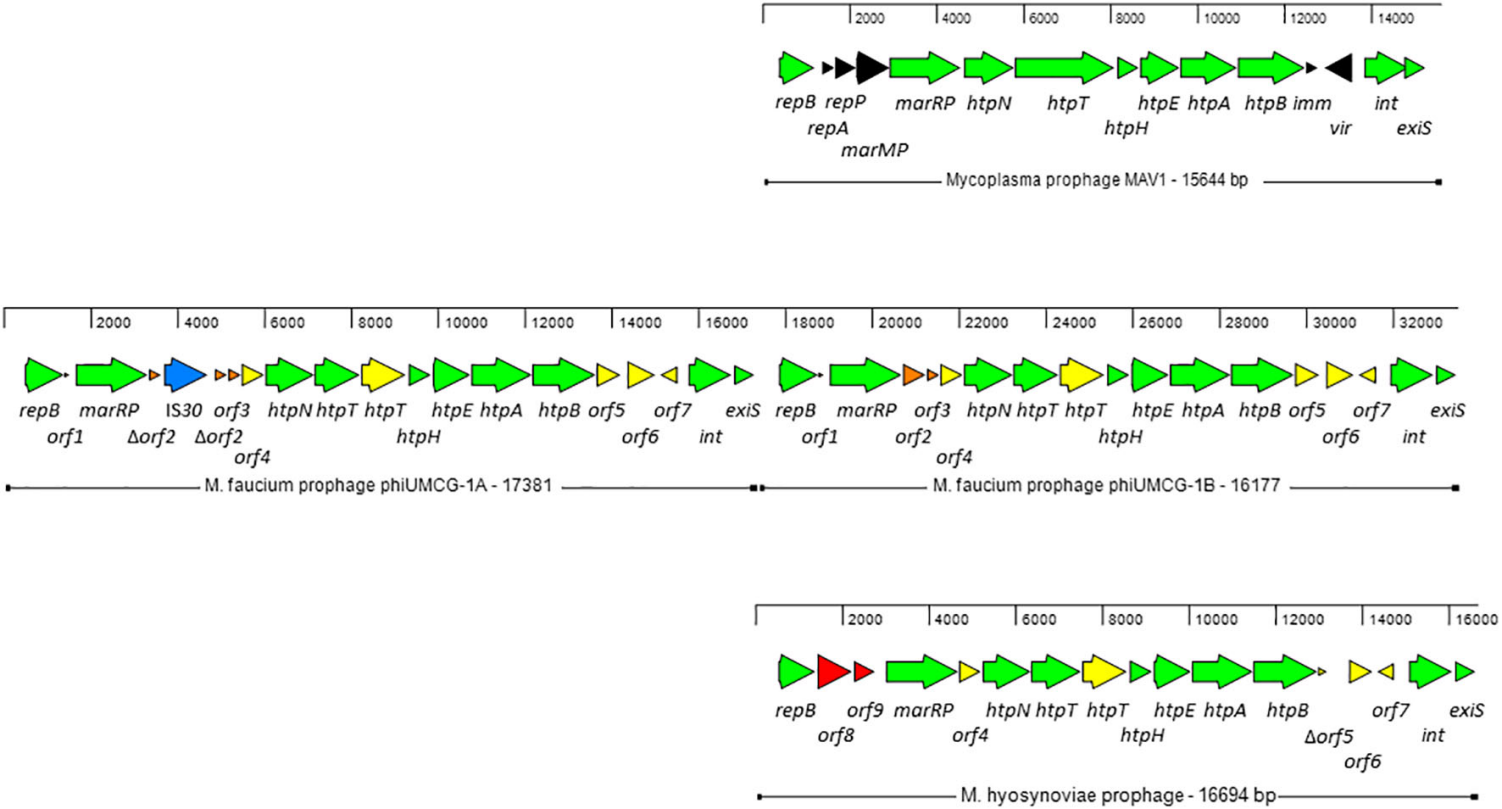
Genome structure of *M. faucium* prophage phiUMCG-1 and comparison with *M. arthritidis* MAV1 (GenBank accession number: NC_001942) and *M. hyosynoviae* MAV1-like (GenBank accession number: CP101129) prophages. The arrows indicate open reading frames and their orientations. Green arrows: genes shared by all prophages. Yellow arrows: genes shared by *M. faucium* and *M. hyosynoviae* prophages phiUMCG-1 and MAV1-like, respectively. Black, orange and red arrows: genes only present in prophages MAV1, phiUMCG-1 and MAV1-like, respectively. Blue arrow: the IS30 gene integrated into *orf2* in one of the copies of phiUMCG-1. Locations and sizes are aligned on a scale showing 2 kb intervals. The genes in *M. faucium* phiUMCG-1 and *M. hyosynoviae* MAV1-like are designated, using the nomenclature published for their counterparts in *M. arthritidis* MAV1 (Röske et al., 2004), where applicable. The genes and their positions in the *M. faucium* UMCG-MFM1 genome are described further in **Supplementary Table S7**.

Two copies of a prophage, designated phiUMCG-1A and phiUMCG-1B, present in tandem on the UMCG-MFM1 chromosome (**Figure 2**) were identified by the phage search tool, PHASTER (Arndt et al., 2016). phiUMCG-1A was 17381 bp long (genome coordinates 129844.147224) while phiUMCG-1B was 16177 bp long (genome coordinates 147216.163392). The two copies were identical with three exceptions. First, the phiUMCG-1A contained an integrated IS30 element within *orf2* encoding a protein of unknown function. Second, there was a T to G nonsynonymous mutation in phiUMCG-1B resulting in a leucine to tryptophan change at position 9 in *orf6*, encoding a hypothetical protein. Third, phiUMCG-1B contained a 77 bp insertion between the genes *orf7* and *int* which encode a hypothetical protein and a tyrosine-type recombinase/integrase, respectively. The closest match in the PHASTER’s databases to phiUMCG-1 was MAV1 (GenBank accession number: NC_001942), a mycoplasma phage encoding 15 proteins that is required for disease development with *Mycoplasma arthritidis* (Voelker and Dybvig, 1999). phiUMCG-1A/B and MAV1 shared 10 genes in common (**Figure 2**). In addition to these, PHASTER analysis revealed that the phiUMCG-1 prophages contained eight other genes including an extra copy of *htpT* encoding a hypothetical protein with some homology to HtpT of the *Mycoplasma* phage, phiMFV1 (GenBank accession number: NC_005964). Although the phiUMCG-1 prophages displayed a similar genomic structure to that of MAV1 (**Figure 2**), they lacked the genes encoding the RepA and RepP proteins of unknown function, DNA methyltransferase MarMP, putative repressor Imm that maintains lysogeny during infection and the Vir determinant that has been regarded as a virulence factor during rats infection with *M. arthritidis* (Röske et al., 2004). The tBLASTn searches using amino acid sequences of each protein encoded by phiUMCG-1A/B, consistently showed that the closest matches in the NCBI database (15 out of 18 proteins) were found to proteins whose structural genes were located in the *M. hyosynoviae* strain B7 chromosome (GenBank accession number: CP101129). The genome of *M. hyosynoviae* strain B7 had previously been subjected to comparative genomic analysis (Bumgardner et al., 2015). The authors found several prophage genes present within the genome of analyzed strains, including chromosome B1 that showed significant similarity to MAV1 (Bumgardner et al., 2015). Moreover, there were also three proteins (encoded by orf1-orf3) specific to *M. faucium* phiUMCG-1 prophages that did not exhibit any sequence similarity to known entries in the current NCBI database.

### Integrative and conjugative element

**Figure 3:**
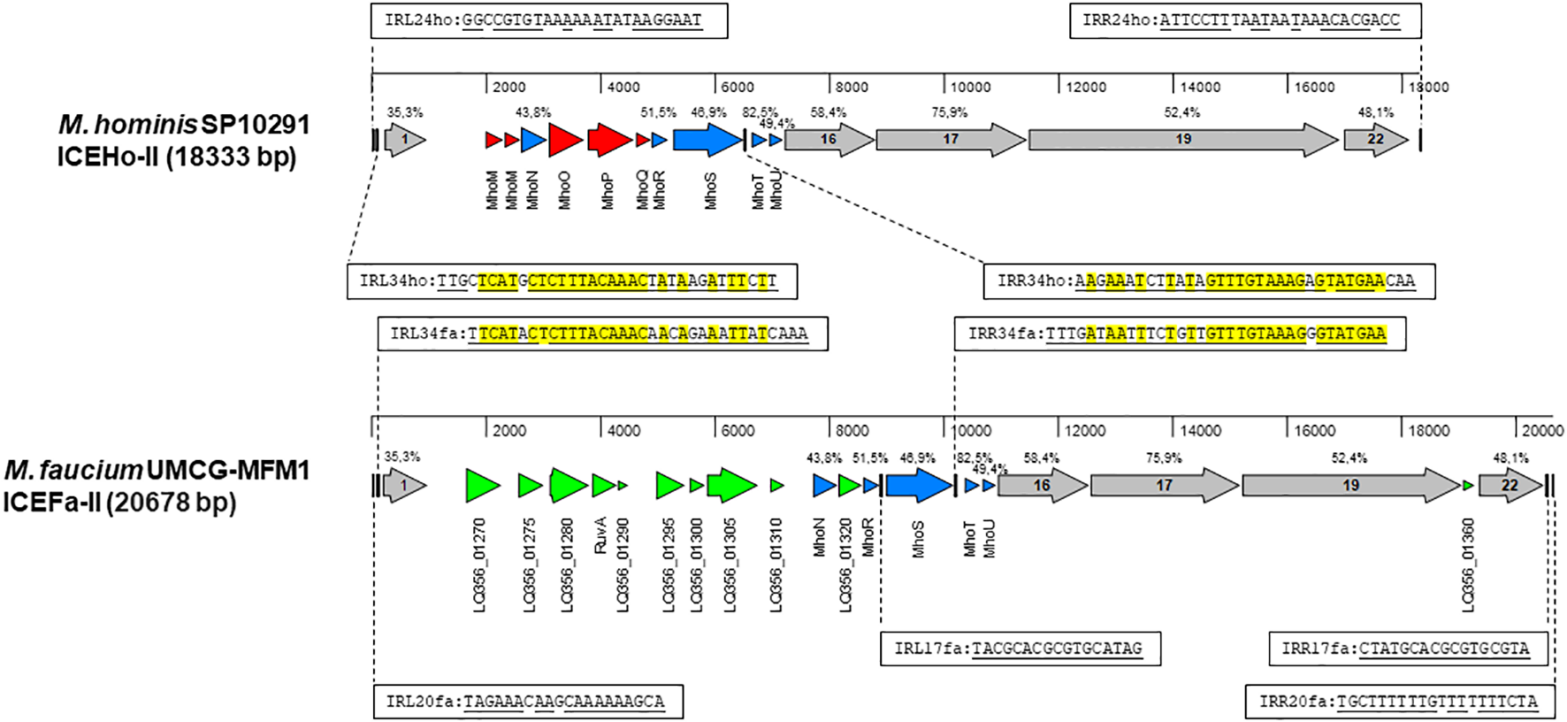
Structural organization of MICE elements. Annotated sequences of the MICE regions were obtained from *M. hominis* SP10291 genome (GenBank accession number: NZ_CP055149, position 13153.31485) and *M. faucium* UMCG-MFM1 genome (GenBank accession number: CP088155) and aligned with MAFFT in MegAlign Pro version 18.0.0(781) (DNASTAR). Sequence identities of homologous proteins, estimated by MAFFT, are shown above the respective genes. A set of MICE core genes, represented by grey arrows, was defined based on a previous analysis of ICEHo-II in *M. hominis* SP10291 (Henrich et al., 2020). The cargo genes shared by both ICEHo-II and ICEFa-II are shown in blue. Genes that are exclusive to ICEHo-II or ICEFa-II are highlighted in red and green, respectively. The inverted repeats are represented by vertical bars. Complementary sequences of the respective inverted repeats are underlined. Nucleotides that are identical in corresponding repeats of both ICEHo-II and ICEFa-II are highlighted in yellow.

There is a growing recognition of the significance of integrative and conjugative elements (ICEs) in horizontal gene transfer (HGT) (Delavat et al., 2017). To identify potential ICE genes in the *M. faucium* genome, we used the protein sequences of all core genes from two different ICE variants, ICEHo-I and ICEHo-II, detected in *M. hominis* genomes (Meygret et al., 2019; Henrich et al., 2020) for tBLASTn searches of the UMCG-MFM1 chromosome. These searches revealed a 20678 bp region (genome coordinates 289110.309787) with a structural organization similar to that of *M. hominis* SP10291 ICEHo-II (**Figure 3**). Based on this homology, we have designated the element as ICEFa-II. Among the 21 annotated genes in the element, protein homology analyses allowed us to classify five of the open reading frames as MICE-core genes CDS-1, −16, −17, −19, and −22, with the encoded proteins exhibiting homologies to the respective ICEHo-II proteins of *M. hominis* SP10291 ranging from 35.3% (CDS-1) to 75.9% (CDS-17). That analysis also identified five of the 10 ICEHo-II non-core (cargo) proteins, MhoN and MhoR to MhoU, as present in ICEFa-II with amino acid identities ranging from 43.8% to 82.5%.

Among the 11 genes in ICEFa-II not found in SP10291 ICEHo-II, PGAP annotation identified two as site-specific DNA methyltransferases: cytosine-specific (LQ356_01280) and adenine-specific (LQ356_01310), two as Holliday junction branch migration protein RuvA and RuvA C-terminal domain-containing protein (LQ356_01290) and seven as hypothetical proteins (**Supplementary Table S6**). Phyre2 analysis of the hypothetical protein sequences revealed that one (locus_tag LQ356_01270) had homology to the DNA-processing protein, DprA, and BLASTp analysis showed the highest homology with DprA of *M. hyosynoviae*. Phyre2 and BLASTp analysis of the remaining six hypothetical proteins did not yield any significant matches.

ICEFa-II was flanked by inverted repeats, IRL20fa and IRR20fa, located 178 bp upstream of CDS1 and 183 bp downstream of CDS 22, respectively. Two additional pairs of inverted repeats were also identified: IRL34fa-IRR34fa and IRL17fa-IRR17fa. Each of these additional pairs of inverted repeats included one terminal repeat, with IRL34fa positioned 137 bp upstream of CDS1 and IRR17fa located 51 bp downstream of CDS 22. These two pairs had internal repeats, IRR34fa and IRL17fa, flanking the *mhoS* gene, positioned 43 bp downstream from the 3’-end and 87 bp upstream from the 5’-end, respectively. Moreover, we identified corresponding sequences for the IRL34fa-IRR34fa pair in ICEHo-II of *M. hominis* SP10291 (**Figure 3**). The presence of these three pairs of inverted repeats suggests that ICEFa-II can be transferred either as a complete unit or as individual components, with each component located between its respective inverted repeats.

### Restriction modification system

To safeguard themselves against invaders such as phages and plasmid DNA, bacteria have evolved a range of protective measures, at least two of which directly target incoming genetic material: the restriction-modification (R-M) system and the clustered regularly interspaced short palindromic repeats (CRISPR)-Cas system.

**Figure 4:**
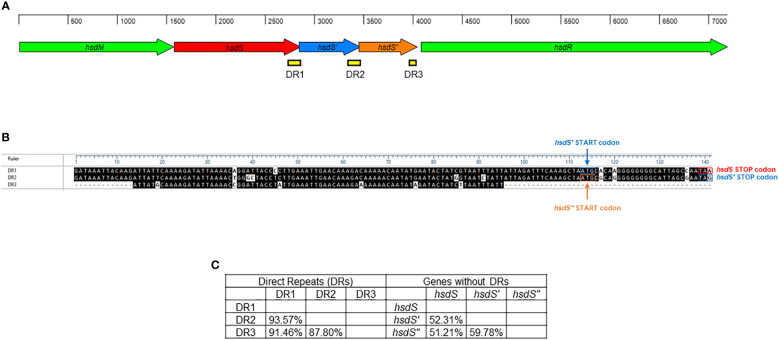
Restriction-modification system of *M. faucium* UMCG-MFM1. **(A)** Structural organization. Arrows represent the genes: *hsdM* (methyltransferase), *hsdS*, *hsdS’*, and *hsdS’’* (specificity subunits), as well as *hsdR* (restriction endonuclease). Direct repeats (DR) are depicted as yellow bars. **(B)** Sequence comparison of DRs. The black shading indicates identical nucleotides within direct repeats. **(C)** Nucleotide sequence identity among analyzed *hsdS* genes and their respective DRs.

A single Type I RM system was identified *M. faucium* UMCG-MFM1 (**Figure 4**), consisting of *hsdM* (encoding the methyltransferase enzyme), *hsdS* (encoding the specificity subunit or DNA recognition domain of the R-M system), and *hsdR* (encoding the restriction endonuclease enzyme). Furthermore, two 5’-terminally truncated *hsdS* pseudogenes, *hsdS’* and *hsdS’’*, were found in tandem downstream of the *hsdS* gene. These pseudogenes contained direct repeat (DR) regions at their 3’-ends. Since the 3’-ends of *hsdS* and *hsdS’* overlapped with the 5’-ends of *hsdS’* and *hsdS’’*, respectively, the 28 bp repeats were also present in the 5’-ends of the *hsdS’* and *hsdS’’* genes. This configuration can enable recombination, potentially leading to the generation of alternative functional *hsdS* variants encoding enzymes with different target specificities. The *hsdS’* and *hsdS’’* pseudogenes exhibited higher identity with each other (59.78%) than when compared to *hsdS* (52.31% and 51.21%, respectively). However, DR1 (located in *hsdS*) showed a higher identity to DR2 (located in *hsdS’*) and DR3 (located in *hsdS’’*), with values of 93.57% and 91.46%, respectively, compared to the identity between DR2 and DR3 (87.80%). This finding strengthens the hypothesis of possible recombination events in the R-M system of UMCG-MFM1.

We found no trace of a CRISPR locus in the UMCG-MFM1 genome. A gene (LQ356_03630) encoding type II CRISPR RNA-guided endonuclease Cas9 was identified although it contained a frameshift mutation generating a premature stop codon expected to produce a non-functional protein.

### Putative virulence factors

Similar to other Mycoplasmas, *M. faucium* UMCG-MFM1 possesses few recognizable genes likely to be involved in virulence. To gain deeper insights into its pathogenic potential, we conducted a comprehensive analysis of the genome using PGAP data analysis, searches using the VFDB protein dataset for *Mycoplasma*, BLASTp against protein sequences deposited in the NCBI database, and PubMed searches. Through these analyses, we identified six potential virulence factors in *M. faucium*, including adherence and invasion proteins, as well as exotoxins (**Table 2**).

**Table 2:** Putative virulence factors of *M. faucium*.

Role in pathogenesis	Gene	Product	Genome position
Adherence	*tuf*	elongation factor Tu	476386.477579
	*gap*	type I glyceraldehyde-3-phosphate dehydrogenase	5284.6288(complement)
Invasion	LQ356_03480	P80 family lipoprotein	763793.765457(complement)
	LQ356_03520	P80 family lipoprotein	773151.775280(complement)
(Exo)Toxin	*vapD*	virulence-associated protein D	18430.18789(complement)
	*hlyA*	TlyA family RNA methyltransferase; Hemolysin A	435744.436475

In pathogenic *Mycoplasma* species, elongation factor Tu (EF-Tu) and type I glyceraldehyde-3-phosphate dehydrogenase (GAPDH) have been identified as the moonlighting proteins (Widjaja et al., 2017; Grimmer and Dumke, 2019). Genes for both proteins were present in *M. faucium* UMCG-MFM1. Besides its primary role in translation, EF-Tu can also interact with host cells and contribute to the bacteria’s ability to adhere to and invade host tissues (Yu et al., 2018). GAPDH has been implicated in adherence to host cells, invasion and modulation of the host immune response (Wang et al., 2023a). It can interact with host cell components and contribute to the bacteria’s ability to establish infection (Wang et al., 2023b).

*M. faucium* UMCG-MFM1 also contained two genes encoding proteins of the P80 family lipoprotein. Both possess a lipoprotein signal peptide, as determined by SignalP-6.0, and exhibit amino acid sequence similarity to other members of the *Mycoplasma* P80 lipoprotein family. Notably, *Mycoplasma agalactiae*, the causative agent of contagious agalactia in small ruminants, produces a P80 protein that is expressed and induces antibody production during the early phase of infection (Tola et al., 1997, Tola et al., 2001).

Certain Mycoplasmas, both human and animal pathogens, demonstrate evident hemolysin activity *in vitro* (Yiwen et al., 2021). Numerous studies have reported on the dual activity of TlyA as both a hemolysin and an RNA methyltransferase (Javadi and Katzenmeier, 2016; Sharma and Singh, 2022). The TlyA family RNA methyltransferase of *M. faucium* (LQ356_01935) exhibited substantial amino acid identity to that of *Mycoplasma* species found in the VFDB protein dataset. *M. faucium* TlyA displayed the highest amino acid identity (55%) with that of *M. pulmonis*, a membrane-associated hemolysin (GenBank accession number AL445566; Gene: MYPU_1710) (Chambaud et al., 2001). The considerable amino acid similarity between the two TlyA proteins suggests a hemolytic activity for *M. faucium*.

### Detection of horizontal gene transfers in the *M. faucium* genome

We identified potentially horizontally transferred genes by examining CDSs in *M. faucium* with BBH in species outside the *M. hominis* phylogenetic group. Out of the 683 annotated CDSs in the *M. faucium* chromosome, 36 exhibited a BBH in a mollicute (35 *Mycoplasma* and 1 *Ureaplasma*), while 27 showed a BBH in a non-mollicute species. We then conducted further examination on each candidate CDS for potential HGT, using phylogenetic reconstructions. We considered evidence of HGT strengthened, especially when a CDS was not identified in any species of the *M. hominis* cluster. After thorough analysis, 9 CDSs were identified that may have been exchanged through HGT (**Table 3**). Notably, two pairs of adjacent CDSs were identified: LQ356_00835 and LQ356_00840, and LQ356_01960 and LQ356_01965. This suggests that the adjacent genes were transferred during the same genetic event. Interestingly, the CDS with locus_tag LQ356_01960, annotated as a Fic family protein, is a part of Toxin-Antitoxin (TA) system. Recently, Hill et al. revealed that HGT is likely to be involved in the distribution of the TA systems in *Mycoplasma* (Hill et al., 2021).

**Table 3:** Horizontal transfer candidates.

*M. faucium* CDS			Closest homolog				Closest homolog in the *M. hominis* cluster		Closest homolog in *Mycoplasma* genus	
Locus_tag	Predicted function	Protein length (amino acids)	Accession number	Species	Identity	Protein cover	Species	Identity	Species	Identity
LQ356_00770	ATP-binding protein	447	MCI5670277	Bacilli bacterium	67.67%	96%	Not found	N/A	*Mycoplasma* sp.	35.68%
LQ356_00815	hypothetical protein	71	HIQ90171	*Candidatus Coprosoma intestinipullorum*	51.43%	98%	Not found	N/A	Not found	N/A
LQ356_00835	AAA family ATPase	572	MBO4339599	Clostridia bacterium	72.76%	54%	*Mycoplasma phocoeninasale*	32.33%	*Mycoplasma anserisalpingitidis*	56.52%
LQ356_00840	LlaJI family restriction endonuclease	417	WP_026399824	*Acholeplasma equifetale*	71.19%	98%	Not found	N/A	*Mycoplasma anserisalpingitidis*	45.13%
LQ356_00855	hypothetical protein	161	WP_013527078	*Mycoplasmopsis fermentans*	92.55%	100%	*Metamycoplasma hominis*	46.43%	*Mycoplasmopsis fermentans*	92.55%
LQ356_01815	hypothetical protein	157	MBX3276393	*Acidobacteriota bacterium*	55.56%	54%	Not found	N/A	Not found	N/A
LQ356_01960	Fic family protein	252	HEN0367640	*Streptococcus agalactiae*	81.25%	95%	*Mycoplasma enhydrae*	27.88%	*Mycoplasma yeatsii*	47.72%
LQ356_01965	hypothetical protein	55	WP_277087801	*Peptostreptococcus porci*	74.07%	98%	Not found	N/A	*Mycoplasma feriruminatoris*	49.06%
LQ356_03265	hypothetical protein	217	OKZ87459	*Clostridium* sp.	70.83%	99%	Not found	N/A	Not found	N/A

N/A – Not Available.

## Discussion

Mycoplasmas typically display organ and tissue specificity (Razin et al., 1998). With the growing number of patients experiencing various types of immunodeficiencies, there is an increasing number of reports regarding the isolation of Mycoplasmas from organs that differ from their usual habitats (Glanville, 2021; Farfour et al., 2024). In recent years, Mycoplasmas have been increasingly documented as the causative agents of post-surgical infections in solid organ transplantation, especially in thoracic surgery, including lung and heart transplantation (Farfour et al., 2024; Tam et al., 2024). In these cases, the overall mortality rate is notably high, which may, in part, be attributed to delays in diagnosing the underlying cause (Farfour et al., 2024). However, the true prevalence of *Mycoplasma* species in lung and heart transplant infections may be underestimated. This underestimation is due to challenges in diagnosis, as the infecting mycoplasmas are slow-growing, have demanding cultivation requirements, or are unculturable. In such cases, identification relies on molecular methods, including genus or species-specific PCR, fluorescence *in situ* hybridization (FISH) assay, 16S rRNA gene sequencing, and whole genome sequencing (Volokhov et al., 2006; Alcorn et al., 2021; Waites et al., 2023). However, those molecular methods are not routinely available (Farfour et al., 2024).

Information about how *M. faucium* phenotypically responds to antibiotics in a controlled laboratory setting is not currently available. Previous attempts to use azithromycin, a macrolide-type antibiotic, in chronic psoriasis did not produce clear outcomes (Saxena and Dogra, 2010). Currently, the sole viable method for predicting the antibiotic susceptibility of *M. faucium* is through genome sequence analysis. Our sequence analysis revealed a limited selection of therapeutic options for addressing *M. faucium* infections, showing solely a possible susceptibility to tetracyclines. Loss of susceptibility to tetracyclines is mediated by *tet* determinants, which are horizontally transferred and prevalent among different *Mycoplasma* species. Therefore, the emergence of fully antibiotic-resistant *M. faucium* strains can be anticipated.

Metagenome-based prediction of antimicrobial resistance (AMR) can achieve high accuracy, contingent upon the volume of bacterial sequence data available. The fundamental concept involves scrutinizing sequencing data for well-known AMR-related genes or single-nucleotide polymorphisms (SNPs) using curated databases such as ResFinder or CARD [76, 77]. The accuracy of this prediction process depends on several factors, including the quantity of sequencing data, the complexity of the sequenced samples, and the level of contaminating host DNA. Despite this, a consensus on the standardized reporting of sequencing depth is currently lacking. The choice of sequencing depth is often influenced by budget constraints and desired outcomes (Greninger, 2018). In our study, the Nanopore reads belonging to *M. faucium* accounted for 0.59% of all reads. Thus the ratio was established at 1 bacterial read for every 169 human reads. Considering the male diploid nuclear genome is approximately 6000.27 megabase pairs (Mbp) and the *M. faucium* genome is 0.806 Mbp, the human genome is approximately 7445 times larger. Assuming that the length of human and bacterial reads in the patient’s sample are comparable in size (mechanical lysis in TissueLyser shears DNA), there was genetic material from approximately forty-four *M. faucium* cells for every one human cell (7445/169). This quantity of *Mycoplasma* material in a human sample was substantial, as a previous report using whole-genome sequencing (WGS) indicated that approximately 0.04% of the total number of unmapped reads to the human genome corresponded to *Mycoplasma* species (Alcorn et al., 2021).

ICEs are horizontally transferred between hosts through type IV secretion systems (T4SS) (Cabezón et al., 2015). Core genes play a pivotal role in the mobilization or conjugation process, while cargo genes often encode specific ICE-associated characteristics such as resistance (Calcutt and Foecking, 2015), metabolic traits (Johnson and Grossman, 2015), or virulence (Leanza et al., 2004). In the case of ICEFa-II, clear identification of resistance or metabolic traits was not evident. However, the presence of low levels of homology poses significant challenges for the bioinformatic analysis of MICE proteins. PGAP annotated two genes in ICEFa-II as encoding DNA methyltransferases: one specific for cytosine (LQ356_01280) and another for adenine (LQ356_01310). Methylation is the primary epigenetic modification of bacterial DNA associated with fitness, defense, and virulence (Seong et al., 2021). In the *M. faucium* genome, other DNA methyltransferases were annotated, including those on a defense island (R-M system), as well as five additional DNA methyltransferases (LQ356_01180, _01375, _01380, _01510, and _02505). Nevertheless, evidence from the analysis of the pan-genome of 233 *Aeromonas veronii* strains suggests that the acquisition of a type I methyltransferase through HGT can increase the drug resistance of that organism (Ma et al., 2023). Comparative genomics and DNA methylation analysis have demonstrated the role of methyltransferases in bacterial pathogenesis (Gao et al., 2023) by likely modifying the host’s genome following infection (Niller and Minarovits, 2016; Pereira et al., 2016; Kot et al., 2020). However, the significance of this finding remains to be fully understood. Future metagenomic investigations on samples recovered from healthy and diseased individuals should cast light on the role of mobile genetic elements and their specific genes in resistance, defense and virulence of *M. faucium*.

The impacts of restriction-modification (R-M) systems on pan-epigenome dynamics and genome plasticity are noteworthy. Notable, Type I R-M systems exhibit phase variability through changes in the number of simple sequence repeats (SSRs) and genetic shuffling of sequences via inverted repeats (IRs) (Atack et al., 2018). Modifications in SSR length located in the HsdS coding sequence lead to ON/OFF switching of DNA methyltransferase activity, potentially altering the specificity of some Type I systems. In *Mycoplasma pulmonis*, phase variation through shuffling by recombination of different *hsdS* genes has been demonstrated (Sitaraman and Dybvig, 1997). This species exhibits DNA inversions involving IRs located in various oppositely oriented *hsdS* genes that can modify the DNA sequence, generating enzyme variants with different DNA binding motifs. In *Mycoplasma agalactiae*, the organization of the *hsd* locus resembles that of *M. pulmonis* (Dordet-Frisoni et al., 2022). The variability of the Type I profile detected in several *M. agalactiae* strains may be explained by potential recombination events. Interestingly, a potential recombinase located within the *M. agalactiae* R-M system may contribute to DNA rearrangement inside the locus. Dordet-Frisoni and colleagues conducted a comprehensive analysis of the genome-wide methylome of *M. agalactiae*, utilizing PacBio SMRT- and Illumina bisulphite-sequencing in conjunction with whole-genome analysis (Dordet-Frisoni et al., 2022). Based on the complete set of DNA methylation patterns within the genomes of *M. agalactiae* strains, the authors of the study analyzed whether each HsdS subunit recognizes a single sequence or whether there is a certain tolerance of the subunits to different sequences. The methylome results indicated that the *hsd* system seems to be capable of accepting certain changes in the target sequence, which may affect its ability to recognize and respond to various sequence variants (Dordet-Frisoni et al., 2022). The identification of tandemly arrayed *hsdS* genes, *hsdS*, *hsdS’*, and *hsdS’’*, in the *M. faucium* UMCG-MFM1 genome (**Figure 4**), all in the same orientation, introduces an intriguing dimension to our study. This arrangement potentially enables recombination and the generation of alternative *hsdS* variants with diverse target specificities, thus changing the epigenomic landscape of *M. faucium*. The observed higher identity between DR1 (located in *hsdS*) and DR2/DR3 (located in *hsdS’*/*hsdS’’*, respectively) supports the notion of recombination events, as evidenced in other Mycoplasmas (Sitaraman and Dybvig, 1997; Dordet-Frisoni et al., 2022). The generation of HsdS enzyme variants with different methylation specificities can be beneficial at a population level during clonal propagation, which may lead to enhanced pathogenesis, better host-adaptation or development of antibiotic resistance (Lee et al., 2022).

To the best of our knowledge, this study represents the first-ever instance of acquiring a complete circularized bacterial genome directly from a patient sample obtained from invasive infection of a primary sterile site using culture-independent, PCR-free clinical metagenomics. The presented approach is a significant advance in both fundamental molecular insights, and clinical decision making in infections with unculturable pathogens.

## Data availability statement

The datasets presented in this study can be found in online repositories. The names of the repository/repositories and accession number(s) can be found in the article/**Supplementary Material**.

## Author contributions

AS: Writing – review & editing, Writing – original draft, Visualization, Validation, Methodology, Investigation, Formal analysis, Data curation, Conceptualization. TD: Writing – review & editing, Software, Methodology, Investigation, Funding acquisition, Formal analysis. SB: Writing – review & editing, Software, Methodology, Formal analysis. VA: Writing – review & editing, Investigation, Formal analysis. AV: Writing – review & editing, Supervision, Formal analysis. AF: Writing – review & editing, Supervision, Formal analysis, Conceptualization. EB: Writing – review & editing, Methodology, Investigation, Formal analysis, Conceptualization.
